# Differential diagnosis of hemangiomas from spinal osteolytic metastases using 3.0 T MRI: comparison of T1-weighted imaging, chemical-shift imaging, diffusion-weighted and contrast-enhanced imaging

**DOI:** 10.18632/oncotarget.20533

**Published:** 2017-08-24

**Authors:** Yan-Jie Shi, Xiao-Ting Li, Xiao-Yan Zhang, Yu-Liang Liu, Lei Tang, Ying-Shi Sun

**Affiliations:** ^1^ Key Laboratory of Carcinogenesis and Translational Research (Ministry of Education), Department of Radiology, Peking University Cancer Hospital & Institute, Beijing 100142, China

**Keywords:** bone tumors, spine, chemical-shift imaging, diffusion-weighted imaging, contrast-enhanced magnetic resonance imaging

## Abstract

The retrospective study investigated accuracy of quantitative evaluation of T1-weighted imaging (T1WI) with and without fat suppression (FS), chemical-shift, diffusion-weighted imaging (DWI) and enhanced imaging at 3.0 T MRI for distinguishing spinal hemangiomas from metastases. 27 patients with 33 spinal hemangiomas (15 atypical hemangiomas) and 26 patients with 71 metastases were recruited. T1WI, FS T1WI, in- and out-phase, DWI and enhanced T1WI were acquired. Signal intensities (SIs) of lesions were obtained. Signal intensity ratios (SIRs) and enhancement ratios of lesions in enhanced imaging were assessed. Ratio of SI loss of hemangiomas or atypical hemangiomas between T1WI and FS T1WI was higher than those of metastases (p < 0.001). The accuracies of ratio of SI loss between T1WI and FS T1WI for differentiating hemangiomas and atypical hemangiomas from metastases were 96.15% and 91.86%. Ratio of SI loss between in- and out- phase could differentiate hemangiomas and atypical hemangiomas from metastases with accuracies of 74.04% and 84.88%. Cutoff values for hemangiomas in SIRs of ≤ 1.52 (early phase) and ≤ 1.38 (middle phase) yielded accuracies of 92.31% and 82.69%. Enhancement ratios of atypical hemangiomas in middle and delayed phases were higher than that of metastases. Accuracies of apparent diffusion coefficient for differentiating hemangiomas and atypical hemangiomas from metastases were 70.19% and 89.53%. T1WI with and without fat suppression could distinguish spinal hemangiomas from metastases. Quantitative assessment of chemical-shift, DWI and enhanced imaging were helpful to identification of spinal hemangiomas and metastases.

## INTRODUCTION

Metastases to spine have been reported to occur in 5-10% of patients with primary neoplasms [1]. A cancer patient undergoing a staging evaluation to detect or rule out bone metastases is important clinically [2]. If metastatic disease is detected, prognosis can change and the treatment regimen can at that point be altered from one of curative therapy to one of palliative treatment [3]. However, some benign spinal lesions may be confused with metastatic lesions and may even be treated as neoplasms unnecessarily using irradiation or chemotherapy [4]. Spinal hemangioma is the most common benign tumor of the spine [5]. An 11% incidence of spinal hemangiomas was reported in an autopsy series in the adult population [6]. Consequently, it is clinically essential to differentiate spinal metastases from hemangiomas in cancer patients.

Magnetic resonance imaging (MRI) has become an important tool for this differentiation between spinal metastases and hemangiomas. Conventional MRI techniques can differentiate typical hemangiomas with hyperintense in relation to the surrounding normal-appearing vertebral bone marrow on T1- and T2-wighted images from bone metastases with hypointense on T1-weighted images and hyperintense or hypointense on T2-wighted images [7]. However, some atypical hemangiomas may vary in MRI appearance including intermediate or hypointense on T1-weighted images [7, 8]. These atypical spinal hemangiomas can mimic bone metastases. The radiologic differential diagnosis between hemangiomas and metastases can be challenging.

During the last years several articles have been published on use of MR sequences like chemical-shift imaging, diffusion-weighted imaging (DWI), and the apparent diffusion coefficient (ADC) in effort to better distinguish a benign vertebral lesion from a malignant one [9–15]. These studies have shown promising results. Our hypothesis is that signal intensity measurements on T1 with and without fat suppression may be the best MR imaging technique to differentiate spinal hemangiomas from metastases. Therefore, the purpose of this study was to investigate the accuracies of quantitative evaluation of T1 with and without fat suppression, chemical-shift imaging, diffusion-weighted and contrast-enhanced imaging at 3.0 T MRI for distinguishing spinal lesions between hemangiomas and metastases.

## RESULTS

From October 2013 to November 2015, 27 consecutive patients with spinal hemangioma including 33 lesions (16 male, 11 female; mean age, 60.62 ± 8.23 years; age range, 41 years to 79 years) who met our criteria were retrospectively recruited from our clinics. Among 33 lesions, 15 were diagnosed as atypical hemangiomas using T1-weighted MR imaging. Of 33 hemangiomas, 12 lesions were 6-12 months follow-up using MR or CT imaging, 7 lesions were 12-24 months follow-up, and 14 lesions with follow-up more than 24 months. 26 consecutive cancer patients with spinal metastases composing 71 lesions (9 male, 17 female; mean age, 54.33 ± 10.66 years; age range, 28 years to 75 years) were also enrolled in the study during the same period. Primary neoplasms included breast cancer (n = 14), rectal cancer (n = 2), colon cancer (n = 1), lung cancer (n = 5), hepatocellular cell carcinoma (n = 2), pancreatic cancer (n = 1), and undetermined primary tumor (n = 1). Of 71 metastatic lesions, 2 were biopsied with pathological confirmation; 25 lesions with metastases were confirmed via 6-12 months follow-up using MR or CT imaging, 19 lesions were 12-24 months follow-up, and 25 lesions with follow-up more than 24 months.

**Figure 1:**
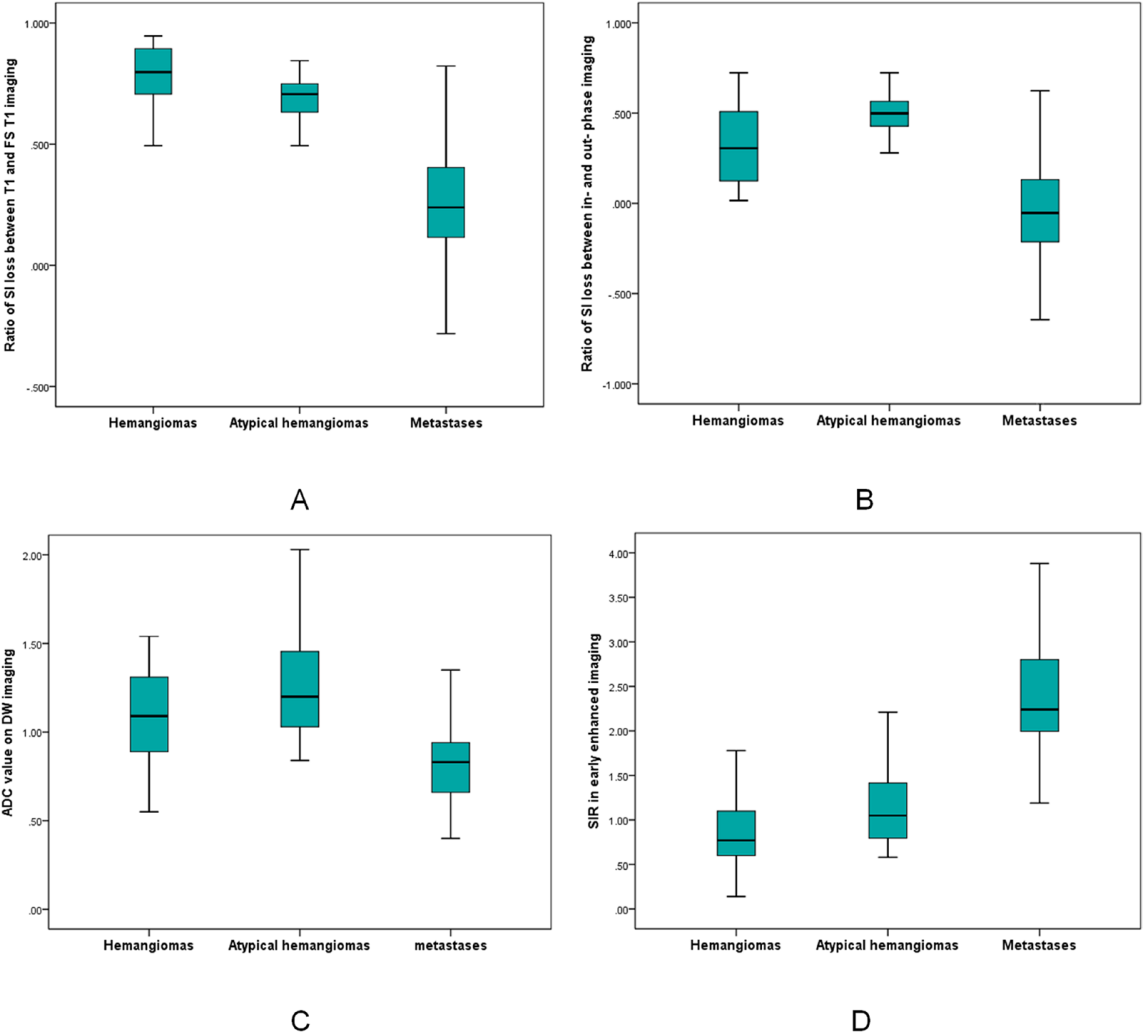
Comparison of hemangiomas, atypical hemangiomas, and metastases **(A-B)** Boxplots summarized value of ratio of SI loss between T1 and FS T1 (A), and in- and out-phase measurements (B). **(C-D)** Boxplots summarized ADC value on DW imaging, and SIR in early enhanced phase.

Quantitative results of ratios of SI loss between T1WI and FS T1WI imaging, and in- and out-phase imaging were summarized in the boxplots shown in Figure 1Aand 3B. The corresponding mean values, SD, as well as statistically significant differences between hemangiomas and metastases were shown in Table 1. Tables 2 and 3 summarized mean values, SD, and statistically significant differences between atypical or typical hemangiomas and metastases. The cut-off values, areas under the curve (AUC), sensitivities, specifities and accuracies of ratios of SI loss between T1 and FS T1 imaging, and in-phase and out-phase imaging for diagnosing hemangiomas, atypical hemangiomas and typical hemangiomas were shown in Tables 4-6, respectively. The ratios of SI loss of hemangiomas or atypical hemangiomas between T1 and FS T1 imaging, and in-phase and out-phase imaging were higher than those of metastases (p < 0.001). The accuracies of SI loss between T1 and FS T1 imaging for diagnosing hemangiomas or atypical hemangiomas were higher than that between in- and out-phase imaging.

**Table 1:** Quantitative assessment in patients with spinal hemangiomas and metastases

Quantitative assessment	Hemangiomas	Metastases	P value
	N = 33	N = 71	
Ratio of SI loss			
T1-WI and FS T1-WI	0.77 ± 0.16	0.24 ± 0.22	< 0.001
In- and out-phase	0.33 ± 0.21	-0.03 ± 0.31	< 0.001
SIR (Early phase)	0.89 ± 0.47	2.56 ± 1.24	< 0.001
SIR (Middle phase)	1.13 ± 0.51	2.13 ± 1.12	< 0.001
SIR (Delayed phase)	1.32 ± 0.59	2.06 ± 0.86	< 0.001
ADC Value (x 10^-3^ mm^2^/s)	1.11 ± 0.36	0.81 ± 0.19	< 0.001

SI, signal intensity; N, number of lesions; FS, fat suppressed; T1WI, T1-weighted imaging; SIR, signal intensity ratio; ADC, apparent diffuse coefficient.

**Table 2:** Quantitative assessment in patients with atypical hemangiomas and metastases in spine

Quantitative assessment	Atypical hemangiomas	Metastases	P
	N = 15	N = 71	
Ratio of SI loss			
T1-WI and FS T1-WI	0.67 ± 0.14	0.24 ± 0.22	< 0.001
In- and out-phase	0.49 ± 0.15	-0.03 ± 0.31	< 0.001
SIR (Early phase)	1.14 ± 0.45	2.56 ± 1.24	< 0.001
SIR (Middle phase)	1.40 ± 0.48	2.13 ± 1.12	< 0.001
SIR (Delayed phase)	1.66 ± 0.50	2.06 ± 0.86	0.085
ADC Value (x 10^-3^ mm^2^/s)	1.24 ± 0.32	0.81 ± 0.19	< 0.001

SI, signal intensity; N, number of lesions; FS, fat suppressed; T1WI, T1-weighted imaging; SIR, signal intensity ratio; ADC, apparent diffuse coefficient.

**Table 3:** Quantitative assessment in patients with astypical hemangiomas and metastases in spine

Quantitative assessment	Typical hemangiomas	Metastases	P
	N = 18	N = 71	
Ratio of SI loss			
T1-WI and FS T1-WI	0.86 ± 0.13	0.24 ± 0.22	< 0.001
In- and out-phase	0.20 ± 0.15	-0.03 ± 0.31	< 0.001
SIR (Early phase)	0.68 ± 0.38	2.56 ± 1.24	< 0.001
SIR (Middle phase)	0.90 ± 0.41	2.13 ± 1.12	< 0.001
SIR (Delayed phase)	1.05 ± 0.50	2.06 ± 0.86	< 0.001
ADC Value (x 10^-3^ mm^2^/s)	1.00 ± 0.37	0.81 ± 0.19	0.048

SI, signal intensity; N, number of lesions; FS, fat suppressed; T1WI, T1-weighted imaging; SIR, signal intensity ratio; ADC, apparent diffuse coefficient.

**Table 4:** Summary of cutoff values, area under curve (AUC) and resulting performance values between spinal hemangiomas and metastases

Quantitative parameter	Cutoff value for hemangiomas	AUC	Sen (%)	Spe (%)	Acu (%)	P value
Ratio of SI loss						
T1- and FS T1-WI	≥ 0.59	0.97 0.94-1.00	90.9%	98.6%	96.15% 100/104	< 0.001
In- and out-phase	≥ 0.06	0.84 0.76-0.91	93.9%	64.8%	74.04% 77/104	< 0.001
SIR (Early phase)	≤ 1.52	0.97 0.94-1.00	90.9%	85.9%	92.31% 96/104	< 0.001
SIR (Middle phase)	≤ 1.38	0.88 0.80-0.95	72.7%	90.1%	82.69% 86/104	< 0.001
SIR (Delayed phase)	≤ 1.62	0.79 0.69-0.89	81.8%	67.6%	71.15% 74/104	< 0.001
ADC Value (x 10^-3^ mm^2^/s)	≥ 0.89	0.78 0.66-0.87	75.8%	67.6%	70.19% 73/104	< 0.001

AUC, areas under the curve; Sen, sensitivity; Spe, specificity; Acu, accuracy; SI, signal intensity; FS, fat suppressed; T1-WI, T1-weighted imaging; SIR, signal intensity ratio; ADC, apparent diffuse coefficient.

**Table 5:** Summary of cutoff values, area under curve (AUC) and resulting performance values between atypical hemangiomas and metastases in spine

Quantitative parameter	Cutoff value for atypical hemangiomas	AUC	Sen (%)	Spe (%)	Acu (%)	P value
Ratio of SI loss						
T1- and FS T1-WI	≥ 0.49	0.95 0.89-1.00	93.3%	93.0%	91.86% 79/86	< 0.001
In- and out-phase	≥ 0.27	0.930 0.88-0.99	93.3%	83.1%	84.88% 73/86	< 0.001
SIR (Early phase)	≤ 1.79	0.95 0.00-1.00	93.3%	85.9%	87.21% 75/86	< 0.001
SIR (Middle phase)	≤ 1.57	0.80 0.70-0.94	80%	78.9%	77.91% 67/86	< 0.001
SIR (Delayed phase)	≤ 1.61	0.66 0.51-0.82	66.7%	67.6%	66.28% 57/86	0.050
ADC Value (x 10^-3^ mm^2^/s)	≥ 1.09	0.89 0.81-0.98	73.3%	93%	89.53% 77/86	< 0.001

AUC, areas under the curve; Sen, sensitivity; Spe, specificity; Acu, accuracy; SI, signal intensities; FS, fat suppressed; T1WI, T1-weighted imaging; SIR, signal intensity ratio; ADC, apparent diffuse coefficient.

**Table 6:** Summary of cutoff values, area under curve (AUC) and resulting performance values between typical hemangiomas and metastases in spine

Quantitative parameter	Cutoff value for typical hemangiomas	AUC	Sen (%)	Spe (%)	Acu (%)	P value
Ratio of SI loss						
T1- and FS T1-WI	≥ 0.68	0.98 0.95-1.00	94.4%	98.6%	97.75% 87/89	< 0.001
In- and out-phase	≥ 0.06	0.76 0.66-0.86	88.9%	64.8%	68.54% 61/89	< 0.001
SIR (Early phase)	≤ 1.18	0.99 0.98-1.00	94.4%	100%	98.88% 88/89	< 0.001
SIR (Middle phase)	≤ 1.32	0.94 0.88-1.00	88.9%	93.0%	92.13% 82/89	< 0.001
SIR (Delayed phase)	≤ 1.25	0.90 0.80-1.00	72.2%	94.4%	89.88% 80/89	< 0.001
ADC Value (x 10^-3^ mm^2^/s)	≥ 0.89	0.67 0.51-0.82	66.7%	67.6%	67.42% 60/89	0.048

AUC, areas under the curve; Sen, sensitivity; Spe, specificity; Acu, accuracy; SI, signal intensities; FS, fat suppressed; T1WI, T1-weighted imaging; SIR, signal intensity ratio; ADC, apparent diffuse coefficient.

The SIRs of hemangiomas, atypical hemangiomas and typical hemangiomas in early phase, middle phase and delayed phase were all lower than those in metastatic lesions (Tables 1-3, Figure 1D). The cut-off values, areas under the curve (AUC), sensitivities, specifities and accuracies of SIRs in early phase, middle phase and delayed phase for diagnosing hemangiomas, atypical hemangiomas and typical hemangiomas were shown in Tables 4-6, respectively.

Overall, there were enhancement ratios of early phase (mean, 2.43; SD 2.10 vs mean, 1.14; SD 0.64, p = 0.001), middle phase (mean, 3.42; SD 2.30 vs mean 1.37, SD 0.65, p < 0.001), and delayed phase (mean, 3.29; SD 2.30 vs mean 1.17, SD 0.60, p < 0.001) in hemangiomas compared with those in metastases. Cutoff values for hemangiomas in enhancement ratios of ≥ 1.65 (early phase), ≥ 2.43 (milddle phase) and ≥ 2.26 (delayed phase) yielded accuracies of 70.2%, 84.6% and 87.5%. There was no significant difference in enhancement ratio of early phase between atypical hemangiomas and metastases (1.75 ± 1.61 vs 1.14 ± 0.64; p = 0.618). There were significant differences in enhancement ratios of middle and delayed phase between atypical hemangiomas and metastases (middle phase, 2.53 ± 1.51 vs 1.37 ± 0.65, p = 0.01; delayed phase, 2.54 ± 1.51 vs 1.17 ± 0.60, p = 0.003, respectively). Cutoff values for atypical hemangiomas in enhancement ratios of ≥ 1.09 (early phase), ≥ 1.94 (milddle phase) and ≥ 1.87 (delayed phase) had accuracies of 61.9%, 77.9% and 83.7%, respectively.

**Figure 2:**
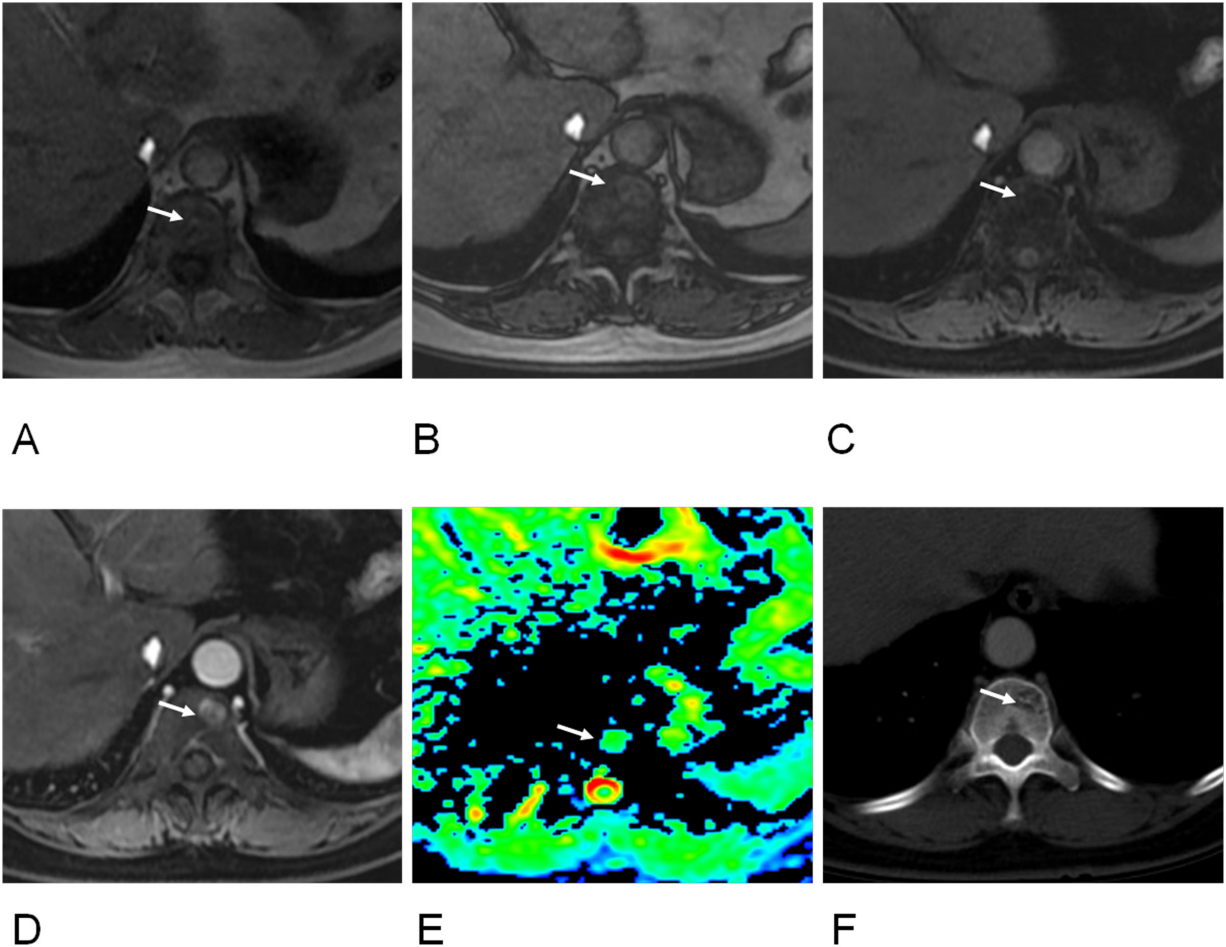
60-year old women with atypical hemangioma of T11 vertebra **(A)** T1-weighted image showed an isointense signal lesion on T11 vertebra (arrow). **(B)** Out-phase image showed a hyperintense signal lesion (arrow), ratio of SI loss between in- and out-phase was 0.35. **(C)** Fat suppressed (FS) T1-weighted images showed an isointense signal (arrow), ratio of SI loss between T1 and FS T1 was 0.74. **(D)** Early enhanced image showed hyperintense signal (arrow) (enhancement ratio, 4.47; SIR, 2.21). **(E)** Apparent diffuse coefficient (ADC) was 1.13 x10^-3^ mm^2^/s (arrow). **(F)** CT image revealed a hypo-dense lesion with internal striated appearance matching with hemangioma (arrow).

**Figure 3:**
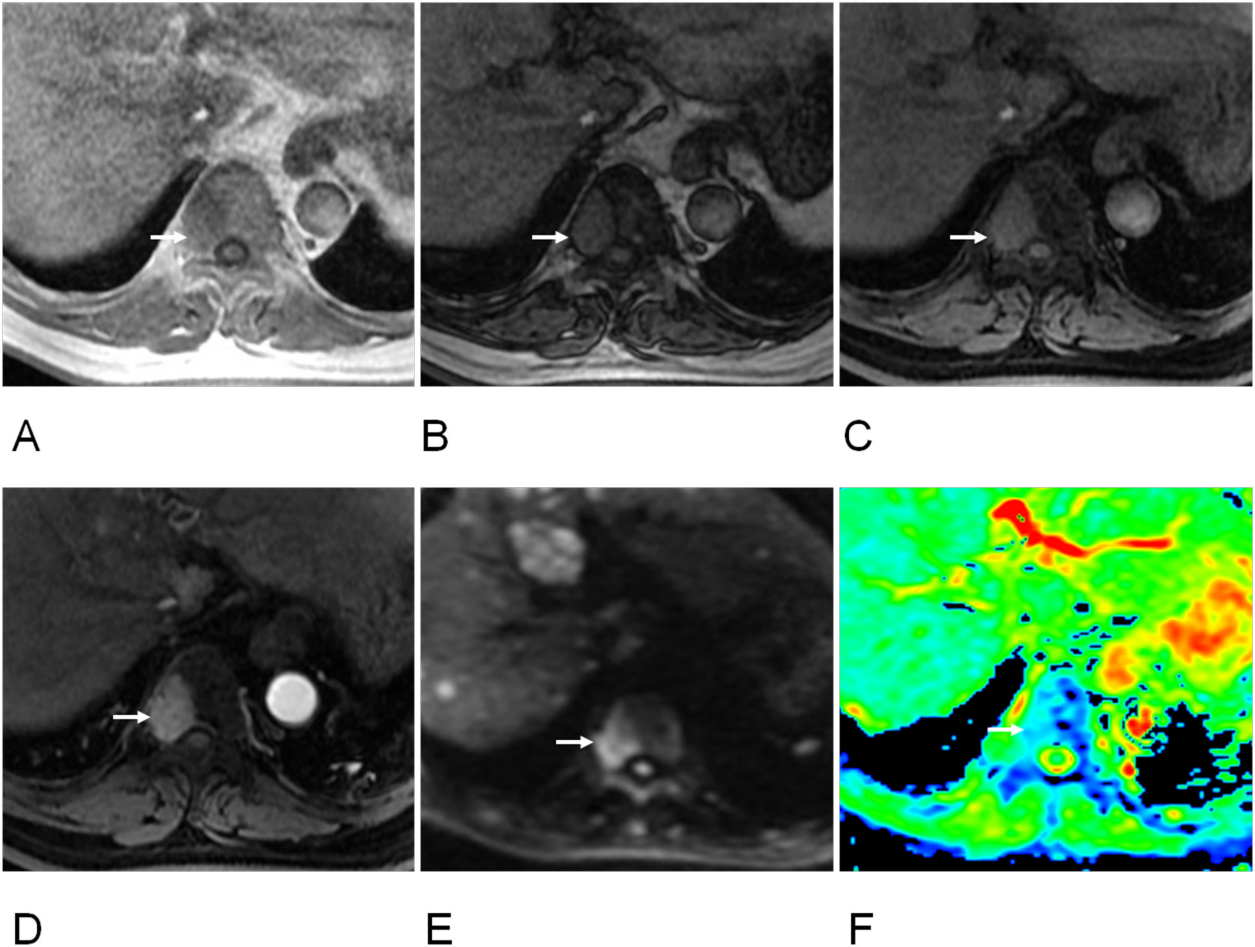
63-year old women of hepatocellular cell carcinoma with metastasis of T9 vertebra **(A)** T1-weighted image showed a hypointense signal lesion on T9 vertebra (arrow). **(B)** Out-phase image showed hyperintense signal (arrow), ratio of SI loss between in- and out-phase was 0.01. **(C)** FS T1-weighted images showed hyperintense signal (arrow), ratio of SI loss between T1 and FS T1 was 0.16. **(D)** Early enhanced image showed hyperintensity (arrow) (enhancement ratio, 0.90; SIR, 4.26). **(E)** DW image showed hyperintensity at b value of 1500 s/mm^2^ (arrow). **(F)** Apparent diffuse coefficient (ADC) was 0.61 x10^-3^ mm^2^/s (arrow).

The ADC values in DW imaging differed significantly between spinal metastases and hemangiomas (Table 1, Figure 1C). The highest accuracy (70.19%) was determined at a cutoff of ≥ 0.89x10^-3^ mm^2^/s for hemangiomas with a sensitivity of 75.8% and a specificity of 67.6% (Table 4). Notably, there was a significant difference in ADC value between metastases and atypical hemangiomas (Table 2, Figure 1C). ROC analysis suggested the use an ADC value of ≥ 1.09 x10^-3^ mm^2^/s to best diagnose atypical hemangioma (accuracy, 89.53%; sensitivity, 73.3%; specificity, 93%) (Figures 2 and 3; Table 5).

## DISCUSSION

Calculating signal intensity (SI) loss between T1 WI and fat suppressed (FS) T1 WI at MRI was able to quantify macroscopic fatty tissue in spinal lesions. Because of no fatty tissue in the metastatic lesions, there was lack of signal loss on fat suppressed imaging compared to non-fat suppressed imaging. Spinal hemangiomas were usually composed of fatty component [8], and a strong signal loss in fat suppressed imaging was expected. Our results showed that the ratio of SI loss of hemangiomas or atypical hemangiomas between T1WI and FS T1WI were higher than those of metastases. Quantitative assessment using ratio of SI lose between T1 and FS T1 imaging was superior to quantitative chemical-shift MRI, quantitative contrast enhanced imaging and ADC values. Ratio of SI loss between T1 and FS T1 imaging had the highest accuracy in differentiating hemangiomas or atypical hemangioma from metastases.

The chemical shift MRI, or in- and out-phase MRI, was often exploited to generate in-phase (IP) and out-phase (OP) images in which the water net magnetization vector was aligned with (IP image) or opposed to (OP image) the fat net magnetization vector, respectively [16]. Chemical-shift MRI was able to quantify microscopic fat tissue. Normal hematopoietic marrow in axial skeleton had microscopic fat component, so a few studies have assessed this technique in spine [12, 13, 15]. If the bone marrow was replaced by metastases, there was lack of signal loss on opposed-phase images [13]. Our hypothesis was that spinal hemangiomas were usually composed of fatty tissue, so a signal loss was also expected. So similar to previous studies, our results showed that SI loss between in- and out-phase in hemangioma was significantly greater than that of metastatic lesions. In our study, accuracy of 74.04% to differentiate hemangiomas from metastases and 84.88% to identification of atypical hemangiomas and metastases were superior to those of other studies that found diagnostic accuracy of 71.7% [13]. However, our result was inferior to those of other studies that showed high sensitivities of 95% and specificities of up to 100% [12, 15]. Our study also showed that accuracy of quantitative assessment of chemical-shift imaging was inferior to that of T1WI with and without fat suppression. It may be due to the presence of macroscopic and microscopic fat within the hemangiomas. The T1WI with and without fat suppression would quantify both fat whereas the in- and opposed-phase sequences measured mainly the microscopic fat more than the macroscopic fat that was clearly visible on T1 sequences.

In our study, the accuracy (92.31%) of SIR in early enhanced phase to differentiating hemangiomas (cut-off value, ≤ 1.52) from metastases was highest than that of other enhanced phases. The spinal metastases with higher SIR in early phase may be associated with rich blood supply of the primary tumor (such as breast and hepatic tumor), which often appeared early enhancement in contrast enhanced imaging. Our results also showed that the accuracy (83.7%) of enhancement ratio in delayed enhanced phase to distinguishing atypical hemangiomas (cut-off value, ≥ 1.87) from metastases was highest than that of other enhanced phases. Atypical hemangioma with delayed enhancement may contribute to a large amount of vascular tissue [8]. SIRs in middle enhanced phase were also accurate enough for differentiating hemangiomas from metastases with accuracy of 82.69% and distinguishing atypical hemangioma from metastases with accuracy of 77.91%. These finding may have clinical implications and contrast enhanced imaging (including early phase, middle phase, and delayed phase) can be applied in daily practice.

The previous authors have described different diagnostic accuracies of ADCs to differentiate malignant skeletal lesions from benign lesions, ranging between 69.6% and 85% [11, 13, 15]. For differentiating of benign and malignant fracture, Geith and colleagues showed a threshold ADC value of 1.48 x 10^-3^ mm^2^/s yielded a highest accuracy of 78.3% [13]. Ahlawat and colleagues found that the highest accuracy (85%) was determined at a threshold minmum ADC value of ≥ 0.9 x 10^-3^ mm^2^/s for differentiating between benign and malignant lesions with a sensitivity of 92% and a specificity of 78% [11]. Martel Villagrán and colleagues revealed that when the lesion had a cutting point of 0.845 x 10^-3^ mm^2^/s for distinguishing benign from malignant vertebral lesions with accuracy of 73% [15]. In our study, the diagnostic accuracy of ADC to distinguishing atypical hemangiomas from metastases was 89.53%, which was higher than that of previous studies. This improvement can be explained by applying different b values in DWI. Our study improved the technical limitations of prior investigations in that multiple (ten) b values and high b values (1500 s/mm^2^) at 3.0 T MRI were used to determine an accurate ADC value. Most previous studies had studied benign population with different types of benign lesions. However, some benign lesions such as cysts or chondroid lesions often had higher ADC values than malignant lesions. Some primary benign lesions with soft tissue tended to have lower ADC values, and these overlapped considerably with the value of malignant lesions [11]. So, our research just selected one type of benign tumor of hemangiomas to research. Our study also found that the ADC values of hemangiomas were significantly higher from metastases. This might be contributed to water molecules within the vascular spaces in hemangiomas [7].

There were several limitations in this study which have to be discussed. Firstly, histological confirmation was not available for all hemangioma and most metastatic lesions, as not every patient with osteolytic metastases or hemangioma had to be treated surgically or had to undergo vertebroplasty. Verification by biopsy was not routinely performed in patients with an apparent hemangioma and metastases. However, clinical and imaging follow-ups were performed in all cases. Secondly, our sample size of atypical hemangioma was relatively small in this study. The results needed to be verified through further studies on larger sample sizes in the future. Thirdly, our study included metastatic lesions from different types of primary tumors. Different tumors had different mixture of tissue elements, water, and blood supply. These differences might lead to variable ADCs, different enhancement ratios and SIRs of metastatic lesions in enhanced imaging.

In conclusion, T1-weighted imaging with and without fat suppression could distinguish hemangiomas from metastases in spine. Quantitative assessment of chemical-shift, diffusion-weighted and enhanced imaging were also helpful to identification of spinal hemangiomas and metastases.

## MATERIALS AND METHODS

### Subject population

This retrospective study was approved by our institutional review board, and a waiver of informed consent was remitted. The entry criteria for patients were following: (1) a history of primary malignancy confirmed by needle biopsy or pathological examination following surgery; (2) patients with spinal lesions who undergone conventional MRI at 3T as well as DWI with ADC values, chemical-shift imaging, and contrast-enhanced imaging; (3) CT scanning on the corresponding vertebrae; (4) ≥ 6 months follow-up with either MR or CT imaging; (5) no radiation and chemotherapy history. Exclusion criteria were the following: (1) spinal lesions complicated with fracture; (2) lesions without a complete MRI examination; (3) lesions of osteoblastic metastases.

### Reference standards

One radiologist with 9 years of experience reviewed all available clinical records on the subjects (the tumor history, patient information, the results of the correlative imaging examinations, and demographic data) and selected subjects with spinal lesions. The observer characterized each spinal lesion as hemangioma or metastasis, based on MRI and CT finding as well as size and number change in the more than 6 months follow-up. Spinal hemangioma was diagnosed by history (when available) or CT or MRI showing its characteristic striated appearance or “polka dot” appearance and follow-up demonstrating radiological and clinical stability of at least 6 months (Figure 2). Hemangioma with hypointense or isointense on T1-weighted images was defined as atypical hemangioma [7, 8]. Spinal metastasis was diagnosed by history (when available) or CT or MRI revealing lytic nature of metastatic lesions or follow-up demonstrating radiological and clinical progression or therapeutic response after anticancer treatment of at least 6 months (Figure 3). More than 6 months follow-up using CT or MRI to the lesions was one of criterions according to the previous studies [6, 13, 17].

### MRI protocol

All examinations were performed at 3.0 T MRI (Discovery MR750, GE Healthcare, Milwaukee, WI, USA). For signal reception, an eight-channel anteroposterior phase-array surface coil covering spinal lesions was placed around the individual. The imaging protocol consisted of unenhanced and enhanced sequences. Unenhanced sequences included: T2-weighted SSFSE (TR, 2000 ms; TE, 199 ms; matrix size, 384x244; slice thickness, 7 mm; inter-slice gap, 1 mm; NEX, 0.54) in axial and coronal planes; fat suppressed (FS) T2-weighted fast spin-echo (FSE) (TR 8000 ms; TE 109 ms; matrix size, 288x256; slice thickness, 7 mm; inter-slice gap, 1mm; NEX, 4) in axial plane, in- and out-of-phase sequences (TR, 3.2 ms; TE, 2 ms and 1 ms; matrix size, 256x192; slice thickness, 4 mm; inter-slice gap, -2 mm; NEX, 1), T1-weighted sequence (TR, 3.2 ms; TE, 2 ms; matrix size, 256x192; slice thickness, 4 mm; inter-slice gap, -2 mm; NEX, 1), fat suppressed (FS) T1-weighted with 3D LAVA-flex sequence (TR, 3 ms; TE, 1.5 ms; matrix size, 256x192; slice thickness, 4 mm; inter-slice gap, -2 mm; NEX, 1) in axial planes. DWI was performed with spin-echo, single-shot echo-planar imaging (EPI) sequence axially acquired prior to contrast administration with gradient factor of b=0, 20, 50, 100, 200, 600, 800, 1000, 1200 and 1500 s/mm^2^ (TR, 6000 ms; TE, 93.3 ms; matrix size, 128x128; slice thickness, 7 mm; inter-slice gap, 2 mm; NEX, 1). The total acquisition time for DW imaging was 6 minutes. Contrast enhanced MRI was performed during early phase (25 s), middle phase (60 s) and delayed phase (120 s) following intravenous injection of gadolinium-diethylenetriamine pentaacetic acid (DTPA) (Magnevist; BayerSchering, Berlin, Germany) at a dose of 0.1 mmol/kg body weight and flow rate 2 ml/s, followed by a 15-ml saline flush. Contrast enhanced MRI was performed using a breath-hold fat-suppressed 3D T1-weighted LAVA sequence (TR, 3.2 ms; TE, 1.5 ms; matrix size, 256x192; slice thickness, 5 mm; inter-slice gap, -2.5mm; NEX, 0.73) in axial planes. The total acquisition time was 30 minutes.

### Imaging analysis

MR images were analyzed by consensus between two experienced radiologists (9 and 8 years experiences in clinical MRI, respectively), working together on a workstation (AW4.6; GE Healthcare). The signal intensities (SIs) of the spinal lesions were qualitatively evaluated on T1-weighted images and described as hypointense, isointense, or hyperintense in relation to the surrounding normal-appearing vertebral bone marrow [13]. The reviewers were blinded to the clinical information and diagnosis. Regions of interests (ROIs) for quantification were defined manually in the lesions using T1WI with and without fat supression, in- and out-phase images, DW images and contrast enhanced T1-weighted LAVA images. A ROI in normal-appearing vertebral bone marrow was also drawn manually in the contrast enhanced T1-weighted LAVA images. All ROIs were selected from the central two-thirds area of lesions in above sequences images. ROIs of normal bone tissues selected from the same vertebra or adjacent vertebral body in enhanced images, and the size were consistent with the lesion ROIs. To ensure the consistency, all measurements were performed three times with consistent size of ROI at different image levels, and average values were calculated. Care was taken to avoid vascular structure and necrotic components. The values of signal intensity (SI) of spinal lesions in T1WI and FS-T1WI, in- and out-of-phase images, and contrast enhanced images were obtained. SIs of normal bone tissues were also obtained in enhanced images. ADC values were calculated on a pixel-by-pixel basis by using built-in software (Functool; GE Healthcare). Ratio of SI loss of spinal lesion between T1- and FS T1-weighted images was evaluated using the following formula: ([SI of lesion in T1 images]-[SI of lesion in FS T1 images])/[SI of lesion in T1 images]. Ratio of SI loss of spinal lesion between in- and out-phase images were calculated using the following formula: ([SI of lesion in in-phase images]-[SI of lesion in out-phase images])/[SI of lesion in in-phase images]. Enhancement ratio was calculated as follows: ([SI of lesion in enhanced images]-[SI of lesion in FS T1 plain images])/[SI of lesion in FS T1 plain images]. Signal intensity ratio (SIR) of lesion in enhanced images was assessed by SI of the lesion dividing SI of normal marrow.

### Statistical analysis

Descriptive statistics were reported. All continuous variables (quantification parameters) were compared for hemangiomas, typical hemangiomas or atypical hemangiomas and metastases in spine by an unpaired two-tailed Student’s t-test. The sensitivities and specifities of the quantitative parameters for the detection of spinal metastases to be differentiated from hemangiomas, typical hemangiomas or atypical hemangiomas were calculated with receiver operating characteristic (ROC) analysis. Cutoff values for the best sensitivity and specificity were given. P values < 0.05 were considered statistically significant. Calculations were performed using SPSS version 22.0 (IBM Corporation, Armonk, NY, USA).
